# Correlation between dynamic contrast-enhanced MRI characteristics and apparent diffusion coefficient with Ki-67-positive expression in non-mass enhancement of breast cancer

**DOI:** 10.1038/s41598-023-48445-2

**Published:** 2023-12-05

**Authors:** Tingting Nie, Mengwei Feng, Kai Yang, Xiaofang Guo, Zilong Yuan, Zhaoxi Zhang, Gen Yan

**Affiliations:** 1 Department of Radiology, Hubei Cancer Hospital, Tongji Medical College, Huazhong University of Science and Technology, No 116 Zhuodaoquan South Load, Hongshan District, Wuhan, 430079 Hubei China; 2 Department of Radiology, the Second Affiliated Hospital of Xiamen Medical College, No 566 Shengguang Road, Jimei District, Xiamen, 361000 Fujian China

**Keywords:** Biomarkers, Cancer, Oncology, Risk factors

## Abstract

As a remarkably specific characteristic of breast cancer observed on magnetic resonance imaging (MRI), the association between the NME type breast cancer and prognosis, including Ki-67, necessitates comprehensive exploration. To investigate the correlation between dynamic contrast-enhanced MRI (DCE-MRI) characteristics and apparent diffusion coefficient (ADC) values with Ki-67-positive expression in NME type breast cancer. A total of 63 NME type breast cancer patients were retrospectively reviewed. Malignancies were confirmed by surgical pathology. All patients underwent DCE and diffusion-weighted imaging (DWI) before surgery. DCE-MRI characteristics, including tumor distribution, internal enhancement pattern, axillary adenopathy, and time-intensity curve types were observed. ADC values and lesion sizes were also measured. The correlation between these features and Ki-67 expression were assessed using Chi-square test, Fisher’s exact test, and Spearman rank analysis. The receiver operating characteristic curve and area under the curve (AUC) was used to evaluate the diagnostic performance of Ki-67-positive expression. Regional distribution, TIC type, and ipsilateral axillary lymph node enlargement were correlated with Ki-67-positive expression (*χ*^2^ = 0.397, 0.357, and 0.357, respectively; *P* < 0.01). ADC value and lesion size were positively correlated with Ki-67-positive expression (*r*_*s*_ = 0.295, 0.392; *P* < 0.05). The optimal threshold values for lesion size and ADC value to assess Ki-67 expression were determined to be 5.05 (AUC = 0.759) cm and 0.403 × 10^–3^ s/mm^2^ (AUC = 0.695), respectively. The best diagnosis performance was the ADC combined with lesion size (AUC = 0.791). The ADC value, lesion size, regional distribution, and TIC type in NME type breast cancer were correlated with Ki-67-positive expression. These features will aid diagnosis and treatment of NME type breast cancer.

## Introduction

Breast cancer has become the most common malignant tumor and the most common cause of cancer death globally^1^. Non-mass enhancement (NME) is a special imaging manifestation of breast cancer. The occurrence rate of NME type breast cancer is comparatively lower than that of mass type, and the detectability of molybdenum target X-ray and ultrasound is limited, albeit its distinctiveness is prominently displayed on dynamic contrast-enhanced magnetic resonance imaging (DCE- MRI)^2^. According to the lexicon for MRI in the breast imaging reporting and data system (BI-RADS)^3^, a non-mass type lesion denotes the occurrence of enhancement in a region of the fibroglandular tissue without space-occupying effect.

The Ki-67 labeling index is considered to be a highly dependable measure of tumor cell proliferation activity^4,5^ and serves as a significant prognostic marker in breast cancer. Many studies have investigated immunohistochemical expression of Ki-67 as a predictive and prognostic marker according to breast cancer molecular subtypes^6–8^. Luminal A subtype (Ki-67 < 14%) demonstrate reduced responsiveness to chemotherapy, while luminal B subtype (Ki-67 ≥ 14%) display response not only to chemotherapy but also to endocrine treatment or molecular-targeted therapy^9^. Additionally, the Ki-67-based preoperative endocrine prognostic index is a viable predictor of relapse risk^10^. Nevertheless, due to the relatively “loose” tissue structure and heterogeneous nature of tumors, the evaluation of Ki-67 expression in needle biopsy samples may not accurately reflect the entirety of the tumor. Hence, there is a clinical need for an accurate and noninvasive predictor of Ki-67 status in breast cancer patients.

Preoperative imaging markers, including DCE-MRI parameters and the apparent diffusion coefficient (ADC) derived from diffusion-weighted imaging (DWI), have the potential to assess cellular proliferation throughout the entirety of the tumor. It has been reported that time intensity curves (TICs) type was highly correlated with Ki-67 expression levels in an evaluation of the correlation of histopathological prognostic factors of DCE-MRI parameters in breast cancer^11^. Previous study has proven that ADC values are correlated with the proliferation marker Ki-67 and histological grade in breast cancer^12^. Choi et al.^13^ reported a correlation between low ADC values and positive expression of Ki-67 in breast cancer patients. Meanwhile, the application of ADC values and TICs combined with Ki-67 in breast cancer have also been reported^12,13^. However, the above studies did not disclose the proportion of cases with NME type breast cancer. At present, the vast majority of studies on NME lesions still focus on predicting benign and malignant or exploring how to reduce unnecessary biopsies. As a highly specific breast cancer on MRI, the relationship between NME type breast cancer and prognosis, such as Ki-67, needs further in-depth study. On the basis of the findings in these previous studies, we questioned whether the Ki-67 status of NME type breast cancer could be predicted preoperatively by using the DCE-based characterization and ADC value.

In the present study, we analyzed the correlation between DCE-based characterization including tumor distribution and internal enhancement pattern, axillary lymph node status, TIC types, ADC values, and lesion size with Ki-67 positive expression in NME type breast cancer. If there are associations between these parameters and Ki-67, they could potentially aid in predicting the outcome and determining treatment in patients of the NME type breast cancer.

## Materials and methods

### Patients

**Figure 1:**
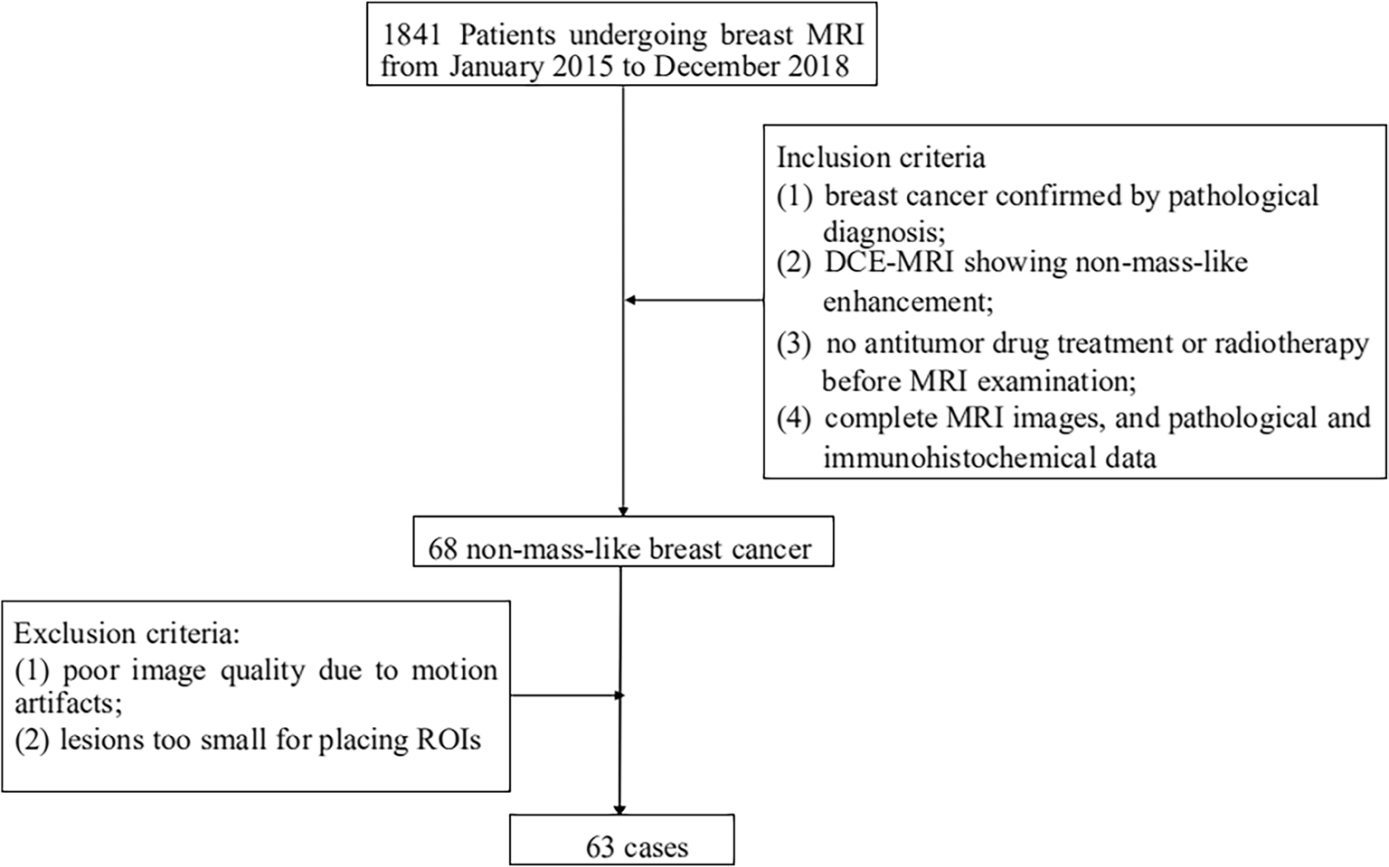
Flowchart of this study.

This retrospective study was approved by the institutional review board of Hubei Cancer Hospital in compliance with ethical principles derived from the Declaration of Helsinki and its subsequent amendments. The data collected for this study are anonymous, and thus the need for obtaining informed consent from participants was waived by the institutional review board of Hubei Cancer Hospital. A total of 1841 patients was retrospectively collected from January 2015 to December 2018. The inclusion criteria were as follows: (a) breast cancer confirmed by surgical pathological diagnosis or puncture biopsy; (b) MRI showing NME, the NME was visually assessed, based on the morphologic characteristics in the T2WI sequence in the axial plane (absence of mass effect) and DCE sequences (presence of interspersed normal parenchyma or fat tissue in the enhancing area); (c) no antitumor drug treatment or radiotherapy before MRI examination; and (d) complete MRI images, and pathological and immunohistochemical data. The exclusion criteria were: (a) poor DWI image quality due to motion artifacts or incomplete fat suppression and (b) lesions too small for placing a region of interest (ROI). In this study, we selected and evaluated a single lesion with the largest diameter per patient, as it is likely that lesions within the same patient would exhibit similar biologic characteristics. Consequently, a total of 63 patients with 63 lesions were included (Fig. 1).

### MR scanning protocol

MR examinations were performed on a 3T MRI scanner (MAGNETOM Verio, Siemens Healthcare, Erlangen, Germany) with an 8-channel breast coil. The sequences were as follows: (1) an axial turbo spin-echo T2-weighted imaging sequence with repetition time/echo time (TR/TE) 5000/61 ms, field of-view (FOV) 320 mm × 320 mm, matrix size 576 × 403, and slice thickness 4 mm; (2) a single shot echo-planar imaging DWI sequence, with b-values 50, 400, and 800 s/mm^2^, TR/TE 7300/83 ms, FOV 320 mm × 110 mm, matrix size 192 × 192, and slice thickness 4 mm; (3) a dynamic series, consisting of a three-dimensional transverse fast low-angle shot T1-weighted sequence with fat suppression, TR/TE 4.66/1.66 ms, FOV 360 mm × 360 mm, flip angle 10, matrix size 384 × 296, and slice thickness 1.2 mm. Gad-opentetate dimeglumine (Magnevist; Bayer Healthcare, Berlin, Germany) was administered intravenously as a bolus (0.1 mmol/kg) by a high-pressure syringe with 3.0 ml/s, followed by a 20 ml of saline flush after the pre-contrast acquisition. Images were acquired in one pre-contrast image, and seven post-contrast acquisitions with no gap centered at 21 s within a total of 8 min and 21 s.

### Image analysis

**Figure 2:**
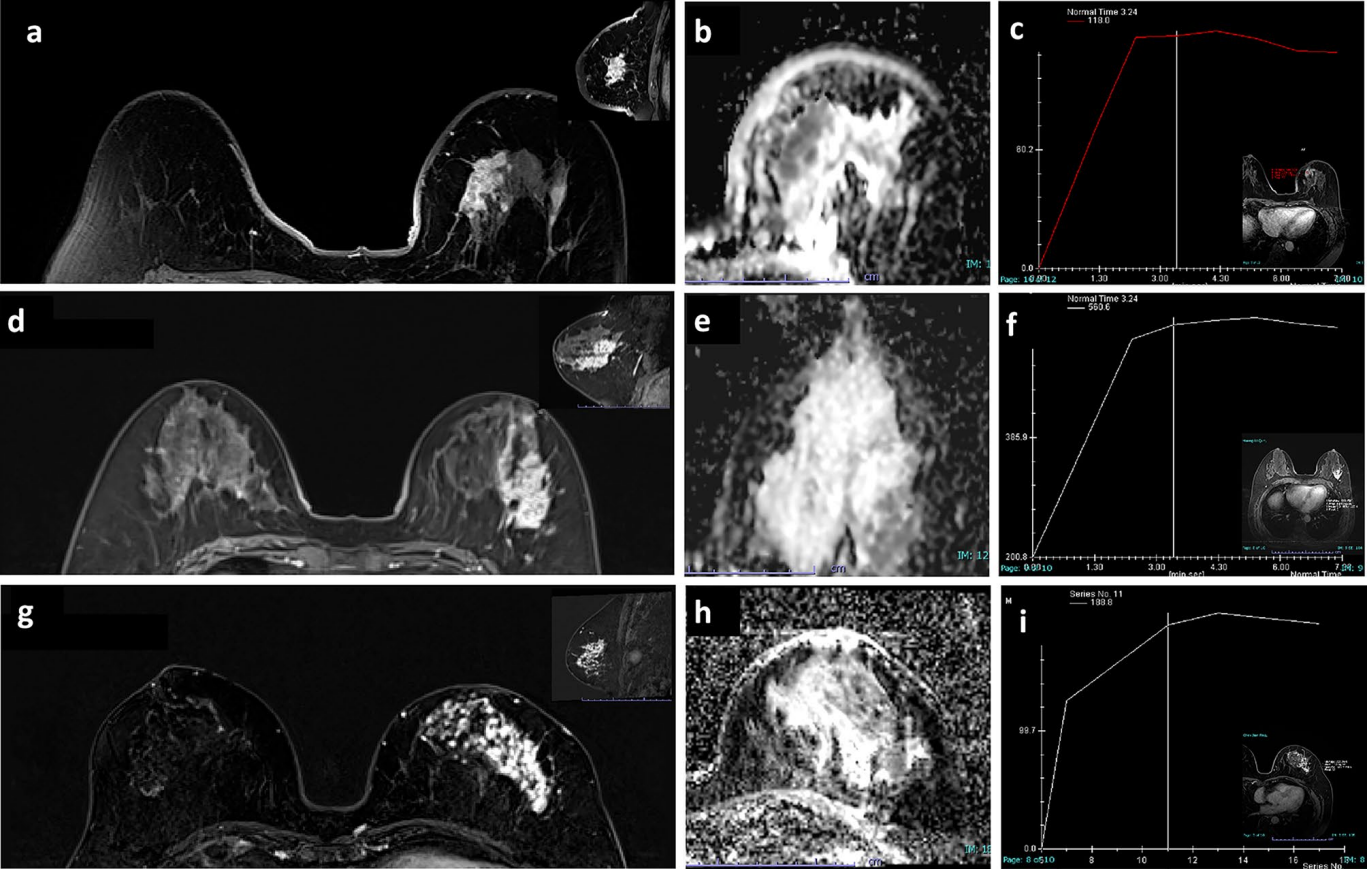
The distribution characteristics of NME lesions. (**a**–**c**) A case of invasive ductal carcinoma. (**a**) The DCE image (axial and sagittal). The lesion displayed a distinct regional pattern, characterized by the presence of geographic enhancement across an extensive region which was not consistent with a singular ductal distribution. (**b**) The ADC image. (**c**) The type II curve was observed. (**d**–**f**) High-grade ductal carcinoma in situ. (**d**) The lesion exhibited a multiple regional distribution, detecting two or more areas of regional enhancement. (**f**) The TIC exhibited a type II pattern. (**g**–**i**) High-grade ductal carcinoma in situ. (**g**) The lesion demonstrating a diffused pattern which were uniformly distributed throughout the breast and exhibited a type II curve (**i**).

**Figure 3:**
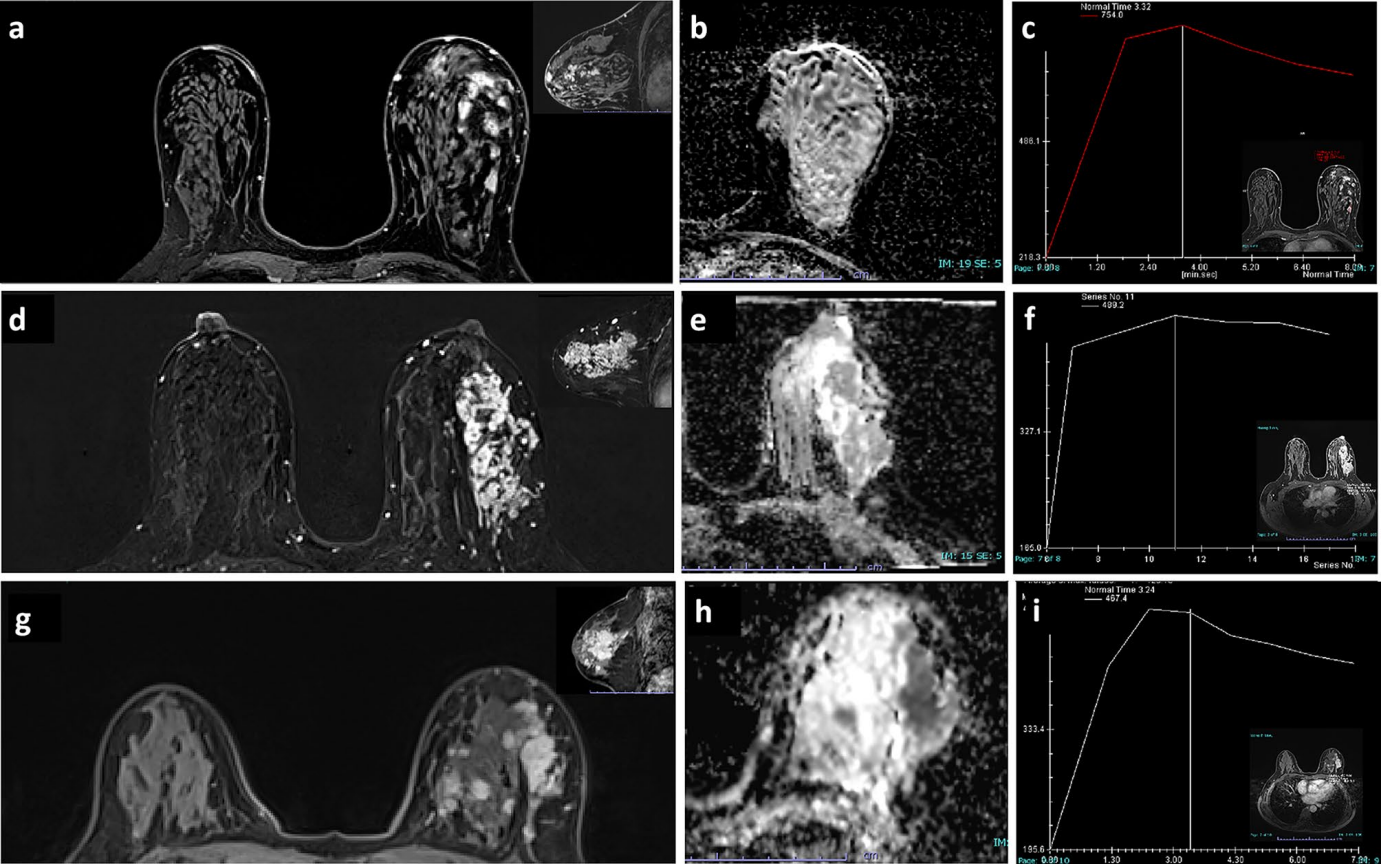
The internal enhancement characteristics of NME lesions. (**a**–**c**) A case of invasive ductal carcinoma (WHO, graded I). (**a**) The DCE image (axial and sagittal). The lesion exhibited a clustered enhancement with uniform size. (**b**) The ADC image. (**c**) The type III curve was observed. (**d**–**f**) Invasive ductal carcinoma (WHO, grade II). (**d**) The lesion displayed a pattern of clustered ring enhancement, indicating the presence of “minute ring enhancements” within an area characterized by heterogeneous NME. (**f**) The TIC exhibited a type II pattern. (**g**–**i**) High-grade ductal carcinoma in situ. (**g**) The lesion demonstrating a heterogeneous enhancement and exhibited a type III curve (**i**).

The internal enhancement and distribution characteristics of NME lesions were evaluated in the DCE and subtraction images (axial, coronal, and sagittal planes) according to the enhancement distribution modifiers and the pattern of internal enhancement (Figs. 2, 3), based on the BI-RADS, 5th edition^14^. The images were evaluated initially by by two radiologists (A and B with 7 and 10 years in breast MR imaging, respectively) blinded to the patients’ clinical details, initial radiological reports, and the pathological results. In case of discrepancy, the findings were jointly reviewed with a third radiologist (C with 15 years of experience in breast MR imaging) in order to achieve a consensus. The tumor size was characterized by the maximum diameter and measured on the reconstructed maximum intensity projection (MIP) image. To mitigate the impact of background parenchymal enhancement (BPE), the assessment of diffused enhancement was recorded in the early phase. For each case, the type of TICs was delineated utilizing DCE-MRI, with a region of interest (ROI) measuring approximately 0.2–0.4 cm^2^ placed on each slice at the brightest section of the lesions observed in the early phase after the contrast injection. Three types of TICs were defined: type I, progressive and continuous enhancement; type II, the early enhancement reached the peak, and the later enhancement increased or decreased by < 10%; type III, rapid enhancement in the early stage, and the enhancement decreased by more than 10% after reaching the peak (Figs. 2, 3). In addition, the criteria for ipsilateral axillary lymph node enlargement were short diameter greater than 1.0 cm or rim enhancement measured on MR enhancement images. The rim enhancement is characterized by a higher signal intensity at the periphery of the node compared to its center^15^.

The MR imaging data for the ADC maps were subsequently transferred to a personal computer and processed employing software (Image J software, version 1.47; http://imagej.nih.gov/ij/). The placement of all ROIs was independently determined by the two readers, based on the DCE images. Both readers assessed the lesions on DW images and ADC maps, recording the mean ADC values on the ADC maps using a 10 mm 2D ROI positioned in the visually darkest region of the enhancing tumor^16^. In case of disparities in the ROI areas, the final review was conducted by the aforementioned third radiologist.

### Immunohistochemical examination

All surgical pathological specimens were subjected to immunohistochemical staining within 1 week, and 100 cancer cells in 10 high-power fields were counted. Positive cells were defined as brownish yellow particles in the cancer nuclei, and the labeling index (LI) was then calculated with the percentage of positive cells in the total number of tumor cells. An LI ≥ 14% was defined as Ki-67-positive and an LI < 14% was defined as Ki-67-negative^9^. Among these patients, 47 patients were in the Ki-67-positive group, and 16 were in the Ki-67-negative group.

### Statistical analysis

Statistical analysis was performed using SPSS software package (SPSS version 22, International Business Machines Corp., Armonk, NY). The Pearson chi-square test and Fisher's exact probability method were used to evaluate the relationship between the distribution characteristics of lesions, internal enhancement characteristics, axillary lymph node enlargement, TIC types, and Ki-67 expression. A Spearman rank correlation analysis was used to calculate the correlation between ADC value, tumor size, a combination of ADC value and lesion size, as well as Ki-67 expression. An ROC curve was used to evaluate the efficacy of above three factors in diagnosing Ki-67 positive expression. The best ADC value and tumor size threshold were obtained according to the Youden index. *P* < 0.05 was considered a statistically significant difference.

## Results

### MR image characteristics

Among 63 patients, 9 had mixed carcinoma, 18 had ductal carcinoma in situ (DCIS), and 36 had invasive ductal carcinoma. The typical DCE-MRI features of NME type breast cancer were diverse. In current study, the internal enhancement characteristics included three categories: clustered (n = 9), clustered ring (n = 31), and heterogeneous (n = 27). For distribution characteristics, 15 patients were regional distribution, 18 were multiple regional distribution, and 27 were diffuse distribution. There were six type I TIC patients, 18 type II, and 36 type III. Only 24 patients had ipsilateral axillary lymph node enlargement.

### Correlation between MR image characteristics and ADC and Ki-67

The Pearson chi square test and Fisher's exact probability method showed that the distribution characteristics of lesions, the type of TIC, and ipsilateral axillary lymph node enlargement were correlated with the positive expression of Ki-67, and the contingency coefficients were 0.397 (*P* = 0.003), 0.357 (*P* = 0.008), and 0.357 (*P* = 0.002), respectively. Although enhancement characteristics of tumors were not correlated with Ki-67 (*P* = 0.082), clustered ring enhancement characteristics accounted for 57.4% (27/47) of findings in the Ki-67-positive group (Table 1).

**Table 1:** Correlation between MR enhanced features and Ki-67 in NME type breast cancer.

DCE-MRI features	Ki-67 positive	Ki-67 negative	χ^2^ value	*P* value
Distribution				
Regional	19.1% (9/47)	37.5% (6/16)	0.397	0.003*
Multiple regional	19.1% (9/47)	56.3% (9/16)		
Diffuse	55.3% (26/47)	6.3% (1/16)		
Internal enhancement				
Clustered	6.4% (3/47)	18.8% (3/16)	0.274	0.082**
Clustered ring	57.4% (27/47)	25.0% (4/16)		
Heterogeneous	38.3% (18/47)	56.3% (9/16)		
TIC type				
I	6.4% (3/47)	18.8% (3/16)	0.357	0.008**
II	25.5% (12/47)	37.5% (6/16)		
III	68.1% (32/47)	25.0% (4/16)		
Ipsilateral axillary lymph node				
Enlargement	48.9% (23/47)	6.3% (1/16)	0.357	0.002**

*The result of Person Chi-square test.

**The result of Fisher's exact probability method.

**Figure 4:**
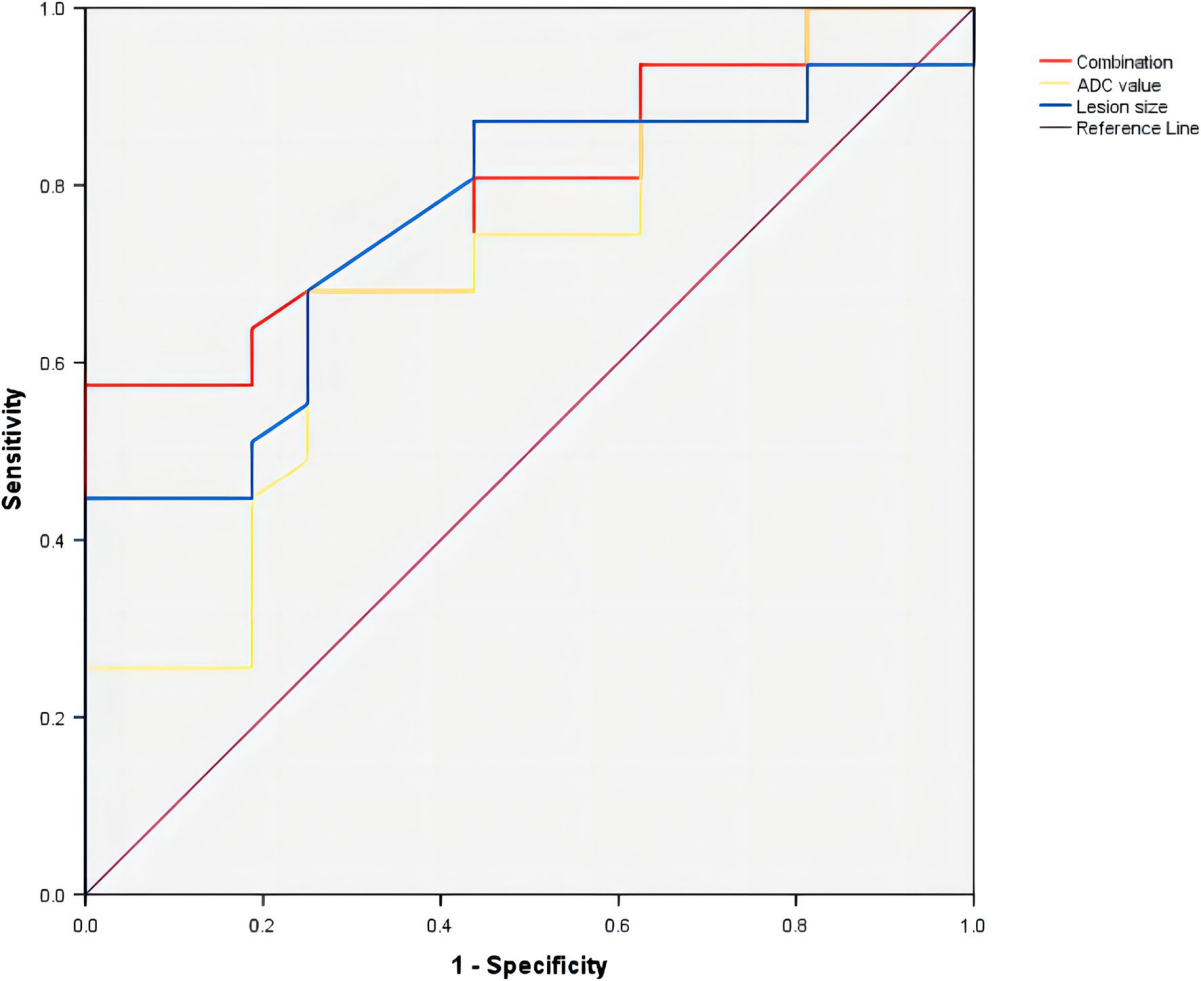
ROC curves of ADC value and lesion size for predicting Ki-67 positivity in non-mass enhancement of breast cancer.

Spearman rank correlation analysis showed that the ADC value and size of lesion were all positively correlated with the positive expression of Ki-67, with *r*_*s*_ values of 0.295 (*P* < 0.05) and 0.392 (*P* < 0.01), respectively (Table 2). Both ADC value and size of lesioncan predict the positive expression of Ki-67, with area under the curve (AUC) values of 0.695 (95% CI: 0.548, 0.843) and 0.759 (95% CI: 0.636, 0.883), respectively. When the ADC value was combined with the lesion size, the diagnostic efficacy reached its maximum with an AUC of 0.791 (95% CI: 0.680, 0.903), indicating the optimal performance (Fig. 4). The Youden index indicated that the cut off values of ADC and tumor size for diagnosis of Ki-67 positive expression were 0.404 s/mm^2^ and 5.05 cm, respectively.

**Table 2:** The correlation between ADC value, lesion size and the positive expression of Ki-67 in NME type breast cancer, and the ROC curve results for predicting Ki-67.

Risk factors	Ki-67		ROC				
	*r*_*s*_	*P value*	Cut-off	Sensitivity (%)	Specificity (%)	AUC value	95% CI
ADC value (s/mm^2^)	0.295	0.019	0.4035	100	18.70	0.695	0.548–0.843
Lesion size (cm)	0.392	0.001	5.05	51.10	81.30	0.759	0.636–0.883
Combination	–	–	–	57.40	100	0.791	0.680–0.903

*ADC* apparent diffusion coefficient, *AUC* area under the curve, *CI* confidence interval, *ROC* receiver operating characteristic.

## Discussion

In this study, we correlated DCE-MRI image characteristics and ADC values of lesions with Ki-67 for NME type breast cancer. Results showed that the distribution characteristics of lesions, TIC types, and ipsilateral axillary lymph node enlargement correlated with the positive expression of Ki-67. The ADC value and tumor size could be used to predict the Ki-67 expression, which is helpful for the monitoring of prognosis and efficacy in NME type breast cancer.

To the best of our knowledge, no previous studies have explored the correlation between the distribution characteristics and internal enhancement features of NME breast cancer and Ki-67. Due to the subjectivity of the BI-RADS descriptors and the issue of inter-observer agreement, we have taken measures to minimize their impact on the results. Previous studies have shown that distinguishing benign and malignant NME lesions or predicting Ki-67 in breast cancer often involves two observers evaluating the imaging features of NME lesions^17–20^. Meanwhile, Lunkiewicz et al.^21^ found that the inter-observer variability and positive predictive value (PPV) for malignancy of NME descriptors, using the fifth edition BI-RADS lexicon, had low disagreement rates for distribution patterns (23.6%) and internal enhancement patterns (22.2%). This noteworthy finding can be ascribed to the utilization of a third impartial radiologist for achieving consensus, in contrast to previous studies that relied on two readers. While there is a dearth of data on inter-observer consistency in other relevant studies, we made the decision to employ a three-observer model to optimize inter-observer consistency.

In this study, we observed a significant association between the distribution characteristics of lesions and the positive expression of Ki-67. Specifically, we found that 45% (26/47) of the patients exhibited a positive expression of Ki-67, suggesting a high proliferative activity of the tumor. Furthermore, these findings indicate that the enhancement characteristics of the diffuse distribution in NME type breast cancer may be attributed to the aforementioned proliferative activity. In terms of internal enhancement characteristics, studies have shown that clustered ring enhancement was significantly correlated with invasive breast cancer and high Ki-67 expression^22^. Ring enhancement on DCE-MRI was associated with the long-term prognosis of triple negative breast cancer patients^23^. Although the findings of this investigation indicate that there is no statistically significant association between internal enhancement characteristics and the positive expression of Ki-67, it is noteworthy that 57.4% (27/47) of lesions in the Ki-67-positive group exhibited clustered ring enhancement characteristics. This observation provides additional support to the earlier statement.

Previous research has utilized TIC analysis for the detection of NME breast cancer. However, Jansen et al.^24^ highlighted the limited diagnostic value of TIC analysis in distinguishing NME breast cancer due to factors such as insufficient blood supply, cases with multi-calcified DCIS, and partial volume effect. Nonetheless, Yang et al.^25^ conducted an analysis of DCE characteristics of NME lesions in conjunction with TIC analysis and semi-quantitative parameters. Their findings demonstrated that the probability of breast cancer in patients with type II curve or dynamic enhancement peak in phase III was significantly higher than that in other types. The sensitivity and specificity of the TIC type in predicting malignant NME lesions were found to be in the range of 94.2% to 96.4% and 29.0% to 58.9%, respectively^17,18^. Similarly, our study revealed that the type of TIC in NME type breast cancer was associated with the positive expression of Ki-67, thus suggesting that TIC classification remains a significant factor in predicting the tumor proliferation status and prognosis of patients diagnosed with NME type breast cancer. This observation may be attributed to the higher likelihood of neovascularization in malignant lesions, resulting in noticeable early enhancement during the DCE early phase. Consequently, it is crucial to position the region of interest (ROI) in the most enhanced areas of the images during the initial stages following contrast injection for the acquisition of TIC types.

The literature has confirmed that ADC values are negatively correlated with positive expression of Ki-67 in breast cancer^26^, but NME type breast cancer is not specifically mentioned. While we found that the ADC value of NME lesions was positively correlated with the positive expression of Ki-67 (*r*_*s*_ = 0.295). One possible reason is that NME type breast cancer contains parts of normal breast tissue with low cell density and limited diffusion of water molecules, so the decrease of ADC value is relatively insignificant, leading to the moderate performance (AUC = 0.695) of ADC in predicting the expression of Ki-67 in NME type breast cancer. Prior research has indicated that using ADC values to identify breast tumors of NME type yields moderate levels of accuracy. Notably, the most favorable outcomes have been achieved through the employment of mean ADC and two-dimensional ROI measurements. Therefore, we adopted a similar approach in this study^16^ Although there are available data indicating high sensitivity (96%) and specificity (100%) of DWI and ADC mapping for evaluating breast lesions, the reproducibility, repeatability, and diagnostic accuracy of DWI in evaluating NME lesions are still under discussion^27–29^. More recently, studies have explored intravoxel incoherent motion and quantitative non-Gaussian diffusion MRI^30–32^. These studies have demonstrated that higher b values might enhance tumor-to-tissue contrast, lesion visibility, and image quality of DWI for detecting and characterizing breast tumors. However, none of these studies have specifically focused on the diagnostically challenging NME lesions, and the potential use of higher b values to improve diagnostic accuracy in NME lesions needs to be further investigated in future studies.

Previous study have demonstrated that the tumor volume of mass breast cancer was smaller than that of NME type breast cancer^33^. In fact, the determination of non-mass lesions is based on their lack of a distinct tumor body, as stated in the definition^3^. Furthermore, research has indicated that the comparison of tumor-pathology size in breast tumors with non-mass enhancements is more prone to overestimation or underestimation compared to masses^34^. In current study, the lesion size of NME type breast cancer was positively correlated with the positive expression of Ki-67 (*r*_s_ = 0.392, AUC = 0.759). Noteworthy, the amalgamation of ADC value and lesion size demonstrated a superior predictive ability (AUC = 0.791) compared to the individual model. This implies that incorporating both lesion size and ADC value could enhance the effectiveness in predicting Ki-67 of NME type breast cancer. Furthermore, the evaluation of axillary lymph node status is considered a crucial prognostic factor that affects the overall disease-free survival in breast cancer patients. However, previous studies have largely overlooked the relationship between ipsilateral axillary lymphadenopathy and positive expression of Ki-67^35^. Our study revealed a correlation between ipsilateral axillary lymphadenopathy and positive expression of Ki-67 (*r*_s_ = 0.392). This provides evidence that patients with ipsilateral axillary lymphadenopathy exhibit highly active tumor proliferation and consequently have an increased risk of metastasis. Furthermore, previous studies have demonstrated a statistically significant correlation between pathologically confirmed axillary lymph node disease and luminal B tumors relative to luminal A tumors (p < 0.01)^36^. These two subtypes exhibit distinct risk profiles and require different treatment strategies. Specifically, Luminal A patients exhibit a more favorable prognosis and primarily receive endocrine therapy, while Luminal B patients necessitate both molecular targeted therapy and chemotherapy in addition to endocrine therapy. Additionally, the expression of Ki-67 is identified as an independent prognostic factor for MFS (Hazard Ratio, 3.27, P = 0.026) and overall survival (HR, 10.64, P = 0.007) in patients with positive axillary lymph nodes^37^. These findings suggest that high Ki-67 expression can serve as a prognostic risk factor in patients with positive axillary lymph nodes and is associated with an inferior prognosis, thus emphasizing the importance of personalized therapy.

There were several limitations in our study. Firstly, the sample size was relatively small and imbalanced, with 47 positive cases and 16 negative cases. However, this is, to the best of our knowledge, the largest cohort of NME breast cancer with Ki-67 reported thus far. Nonetheless, the aforementioned characteristics of the sample size unavoidably increased the risk of bias, necessitating a further expansion of the sample size for more comprehensive research. Secondly, the internal enhancement characteristics of NME type breast cancer may be influenced by the observation phase and orientation. Therefore, our study may have missed some potential NME lesions. Moreover, due to the limited sample size and the inherent characteristics of malignant NME lesions, our cases did not include lesions with segmental or linear distribution. Thirdly, a previous study found relatively low inter-observer variability regarding the evaluation of malignancy of NME descriptors when assessed by consensus among three independent radiologists^21^. Although we followed a similar approach, potential inter-reader variability has not been completely eliminated. Further research is necessary to confirm the repeatability of inter- and intra-observer evaluations.

In conclusion, multiple imaging characteristics of NME type breast cancer defined by the fifth edition of the BI-RADS MRI dictionary, including lesion distribution, internal enhancement, ipsilateral axillary lymph node enlargement, ADC value, and lesion size were correlated with the positive expression of Ki-67. The results of this study can indirectly provide a reference for the differential diagnosis, treatment, and prediction of prognosis of patients with NME type breast cancer.

## Data Availability

The datasets used and/or analysed during the current study available from the corresponding author on reasonable request.

## References

[1] Sung H, Ferlay J, Siegel RL (2021). Global cancer statistics 2020: GLOBOCAN estimates of incidence and mortality worldwide for 36 cancers in 185 countries. CA Cancer J. Clin..

[2] Li X, Cheng L, Liu M (2013). Comparative study of MRI, mammography and ultrasonography in the diagnosis of breast diseases presented as non-mass like enhancement. Chin. J. Med. Imaging.

[3] Spak DA, Plaxco JS, Santiago L (2017). BI-RADS® fifth edition: A summary of changes. Diagn. Interv. Imaging.

[4] Onishi N, Kanao S, Kataoka M (2015). Apparent diffusion coefficient as a potential surrogate marker for Ki-67 index in mucinous breast carcinoma. J. Magn. Reason. Imaging.

[5] Davey MG, Hynes SO, Kerin MJ (2021). Ki-67 as a prognostic biomarker in invasive breast cancer. Cancers.

[6] Wei XX, Zhang R, Pu TJ (2017). Ki-67 expression and its effect on response to neo-adjuvant chemotherapy in invasive breast cancer. Zhonghua Bing Li Xue Za Zhi.

[7] Soliman NA, Yussif SM (2016). Ki-67 as a prognostic marker according to breast cancer molecular subtype. Cancer Biol. Med..

[8] Chen X, He C, Han D (2017). The predictive value of Ki-67 before neoadjuvant chemotherapy for breast cancer: A systematic review and meta-analysis. Future Oncol..

[9] Goldhirsch A (2011). Strategies for subtypes—dealing with the diversity of breast cancer: Highlights of the St. Gallen international expert consensus on the primary therapy of early breast cancer 2011. Ann Oncol..

[10] Ellis MJ, Suman VJ, Hoog J (2017). Ki67 proliferation index as a tool for chemotherapy decisions during and after neoadjuvant aromatase inhibitor treatment of breast cancer: Results from the American College of Surgeons Oncology Group Z1031 Trial (Alliance). J. Clin. Oncol..

[11] Tuan LL, Minh DN, Tra MT (2021). Correlations between dynamic contrast-enhanced magnetic resonance imaging parameters and histopathologic factors in breast cancer. Clin. Ter..

[12] Tuan LL, Minh DN, Minh DN (2021). Correlations between apparent diffusion coefficient values and histopathologic factors in breast cancer. Clin. Ter..

[13] Choi SY, Chang YW, Park HJ (2012). Correlation of the apparent diffusion coefficiency values on diffusion-weighted imaging with prognostic factors for breast cancer. Br. J. Radiol..

[14] Marino MA, Avendano D, Zapata P, Riedl CC, Pinker K (2020). Lymph node imaging in patients with primary breast cancer: Concurrent diagnostic tools. Oncologist.

[15] Mendelson, E. B. *et al*. ACR BIRADS® Ultrasound. In *ACR BI-RADS® Atlas, Breast Imaging Reporting and Data System* (American College of Radiology, 2013).

[16] Avendano D (2019). Limited role of DWI with apparent diffusion coefficient mapping in breast lesions presenting as non-mass enhancement on dynamic contrast-enhanced MRI. Brest Cancer Res..

[17] Liu G (2022). Non-mass enhancement breast lesions: MRI findings and associations with malignancy. Ann transl. Med..

[18] Li Y (2020). Non-mass enhancements on DCE-MRI: Development and validation of a radiomics-based signature for breast cancer diagnoses. Front. Oncol..

[19] Soylu Boy FN, Esen Icten G, Kayadibi Y (2023). Idiopathic granulomatous mastitis or breast cancer? A comparative MRI study in patients presenting with non-mass enhancement. Diagnostics.

[20] de Faria Castro Fleury E (2023). Management of non-mass enhancement at breast magnetic resonance in screening settings referred for magnetic resonance-guided biopsy. Breast Cancer.

[21] Lunkiewicz, M. *et al*. Interobserver variability and likelihood of malignancy for fifth edition BI-RADS MRI descriptors in non-mass breast lesions. *Eur. Radiol*. **30**(1),77–86 (2020). 10.1007/s00330-019-06312-7 PMC6890614 31392476

[22] Mercado CL (2014). BI-RADS update. Radiol. Clin. North Am..

[23] Lee, S. M. *et al*. Patterns of malignant non-mass enhancement on 3-T breast MRI help predict invasiveness: Using the BI-RADS lexicon fifth edition. *Acta Radiol*. **59**(11), 1292–1299 (2018). 10.1177/0284185118759139 29758996

[24] Jansen SA, Fan X, Karczmar GS (2008). DCEMRI of breast lesions: Is kinetic analysis equally effective for both mass and nonmass-like enhancement?. Med. Phys..

[25] Yang QX, Ji X, Feng LL (2017). Significant MRI indicators of malignancy for breast non-mass enhancement. J. Xray Sci. Technol..

[26] Aydin H, Guner B, Esen BI (2018). Is there any relationship between adc values of diffusion-weighted imaging and the histopathological prognostic factors of invasive ductal carcinoma?. Br. J. Radiol..

[27] Rahbar H, Zhang Z, Chenevert TL (2019). Utility of diffusion-weighted imaging to decrease unnecessary biopsies prompted by breast MRI: A trial of the ECOG-ACRIN cancer research group (A6702). Clin. Cancer Res..

[28] Spick C, Bickel H, Pinker K (2016). Diffusion-weighted MRI of breast lesions: A prospective clinical investigation of the quantitative imaging biomarker characteristics of reproducibility, repeatability, and diagnostic accuracy. NMR Biomed..

[29] An YY, Kim SH, kang BJ (2017). Differentiation of malignant and benign breast lesions: Added value of the qualitative analysis of breast lesions on diffusion weighted imaging (DWI) using readout-segmented echo-planar imaging at 3.0 T. PLoS ONE.

[30] Iima M, Kataoka M, Kanao S (2018). Intravoxel incoherent motion and quantitative non-Gaussian diffusion MR imaging: Evaluation of the diagnostic and prognostic value of several markers of malignant and benign breast lesions. Radiology.

[31] Bickel H, Polanec SH, Wengert G (2019). Diffusion-weighted MRI of breast cancer: Improved lesion visibility and image quality using synthetic b-values. J. Magn. Reason. Imaging.

[32] Goto M, Le Bihan D, Yoshida M (2019). Adding a model-free diffusion MRI marker to BI-RADS assessment improves specificity for diagnosing breast lesions. Radiology..

[33] Liu H, Peng W (2012). MRI morphological classification of ductal carcinoma in situ (DCIS) correlating with different biological behavior. Eur. J. Radiol..

[34] Mann RM, Bult P, van Laarhoven HWM (2013). Breast cancer size estimation with MRI in BRCA mutation carriers and other high risk patients. Eur. J. Radiol..

[35] Liu Q, Wang XZ, Mu DB (2015). Correlation analysis of hormone receptors and the expressions of HER-2 and Ki-67 in breast cancer. Eur. J. Gynaecol. Oncol..

[36] Plaza MJ, Handa P, Esserman LE (2017). Preoperative MRI evaluation of axillary lymph nodes in invasive ductal carcinoma: Comparison of luminal A versus luminal B subtypes in a paradigm using Ki-67 and receptor status. AJR Am. J. Roentgenol..

[37] Li FY (2017). Prognostic value of Ki-67 in breast cancer patients with positive axillary lymph nodes: A retrospective cohort study. PLoS ONE.

